# Kawasaki disease recurrence in the COVID-19 era: a systematic review of the literature

**DOI:** 10.1186/s13052-021-01041-4

**Published:** 2021-04-19

**Authors:** Alice Annalisa Medaglia, Lucia Siracusa, Claudia Gioè, Salvatore Giordano, Antonio Cascio, Claudia Colomba

**Affiliations:** 1 Department of Health Promotion, Mother and Child Care, Internal Medicine and Medical Specialties, Infectious Diseases Unit, University of Palermo, Palermo, Italy; 2 ARNAS Civico-Di Cristina, Pediatric Infectious Diseases Unit, Palermo, Italy

**Keywords:** Recurrent Kawasaki disease, Kawasaki disease recurrence, Covid-19, Sars-Cov2

## Abstract

Kawasaki disease (KD) is a vasculitis of unknown origin of small and medium caliber blood vessels, especially involving coronary arteries and is the leading cause of acquired heart disease in childhood in developed countries. Although rarely, it can recur: most recurrences occur within 2 years of the initial episode. No data are available on incidence of recurrent KD in Europe and multiple recurrences are rarely seen. We reviewed the medical literature on Kawasaki disease recurrence and reported a new case of Kawasaki disease recurrence in a child with SARS-CoV-2 infection. We believe that in our case SARS Cov2 acted as a trigger capable to determine, in a genetically susceptible individual, a second recurrence of the disease. In the Covid-19 era we affirm the importance for Kawasaki disease to be tested for SARS Cov2 infection.

**Supplementary Information:**

The online version contains supplementary material available at 10.1186/s13052-021-01041-4.

## Main text

### Background

Kawasaki disease (KD) is a vasculitis of unknown origin of small and medium caliber blood vessels, especially involving coronary arteries. It mainly affects children under 5 years of age and, in developed countries, represents the leading cause of acquired heart disease in childhood. Coronary artery abnormalities (CAAs) occur approximately in 25% of untreated patients. Diagnosis is based on the presence of fever (lasting > 5 days) and of 4 of the 5 classic diagnostic criteria (oropharyngeal changes, bilateral bulbar conjunctival injection without exudate, rash, change of the extremities, cervical lymphadenopathy ≥1.5 cm diameter). However, diagnosis can be made with incomplete features if fewer than 4 of the above-mentioned criteria are present. Despite efforts over 40 years, the etiology and pathophysiology of KD are still unknown. It is generally thought that KD results from a variety of infectious agents that evoke an abnormal immunologic response in genetically susceptible individuals [1]. An episode of complete or incomplete KD that occurs after the complete resolution of the previous episode is defined recurrent KD. Most recurrences occur within 2 years of the initial episode. If the recurrency occurs before than 2 months from first episode, it can also be defined recrudescence [2] Recurrence rate varies from region to region and closely follows the trend of incidence of KD [3]. No data are available on incidence of recurrent KD in Europe and multiple recurrences are rarely seen [4] Recent evidence reported that Severe acute respiratory Syndrome Coronavirus 2 (Sars-Cov-2) can causes an inflammatory syndrome whose pattern of overexpressed cytokines overlaps with that typical of KD [5]. There is growing concern of SARS-CoV-2 infection related inflammatory syndrome as a possible link between coronavirus infection and KD affecting young children [6] Clinical reports have recently been published from the United States [7, 8], Italy [9], the United Kingdom [10], France and Switzerland [11], all describing a new COVID-19 related clinical syndrome, with significant inflammation and similarities to KD [12]. Here we report the first case of KD recurrence in a child with SARS-CoV-2 infection and made a systematic review of the literature to describe the epidemiologic and clinical characteristics of KD recurrence.

### Case report

A 3-year-old Caucasian boy with a previous diagnosis of recurrent KD was admitted for 5-day fever (maximum 39 °C), painful feet’s edema, maculopapular exanthema, and cheilitis. Indeed, 21 and 11 months before current admission (at the age of 20 and 29 months) he was respectively diagnosed as typical KD and recurrent KD, successfully treated with intravenous immunoglobulin (IVIG) 2 g/Kg followed by aspirin, and no CAA was diagnosed.

At the admission, he had a temperature of 39.7 °C, diffuse maculopapular exanthema, cheilitis, hyperemic pharynx, cervical lymphadenopathy, edematous tongue, conjunctival injection, hyperemic genital and anal mucosa, and painful feet’s edema. Neither joint nor muscle pain were present at admission. Family history was negative for autoinflammatory or genetic syndromes. The C-reactive protein level was 140 mg per liter, ESR was 49 mm/h, white-cell count was 13,400 per cubic millimeter, platelet count 502,000 per cubic millimeter, fibrinogen level was 6.2 g/l (normal range, 2 to 4), LDH, AST/ALT, ferritin, troponin T, pro-BNP, the prothrombin and activated partial thromboplastin times were normal. Autoimmune workup, including peripheral blood lymphocyte subtypes assay, Immunoglobulin classes assay, Complement activation (C3, C4) assay and serum lipid profile, was negative. Transthoracic echocardiography showed no CAAs. Nasopharyngeal swab for SARS-Cov-2, blood culture, nasal swab for viruses, serology for Mycoplasma, Chlamydia, CMV, EBV, parvovirus B 19 were performed. As all diagnostic criteria of KD were present, IVIG were immediately administered at a dose of 2 g per kilogram of body weight, followed by acetylsalicylic acid at a daily dose of 50 mg per kilogram of body weight. Fever disappeared 24 h after, exanthema slowly vanished, feet’s edema reduced, ulcerative oral lesions improved, C-reactive protein, ESR and white-cell count progressively decreased. All microbiological exams were negative, except for SARS-CoV2 PCR on nasopharyngeal swab which gave a positive result. Chest X-ray showed slight hilar interstitial infiltrates. IL-6 was 43 pg/ml (normal range, 0 to 16.4). Nasopharyngeal swabs performed 7 and 9 days later yielded a negative result. The child was discharged and he is currently fine and on rheumatological follow up.

## LITERAUTRE review

### Materials & methods

A computerized search was performed without language restriction using PubMed and Scopus, combining the terms (Kawasaki disease OR Recurrent Kawasaki disease) AND (children OR child OR infant OR paediatric), with no filters. Furthermore, all references listed were hand-searched for other relevant articles. An article (both case reports and case series) was considered eligible for inclusion if it reported single or multiple recurrence (or recrudescence) of KD in children, as long as data on patients were clearly extractable either from abstract, article, or tables. The following epidemiologic and clinical variables were evaluated for each case: sex, age, clinical features, number of recurrences, time elapsed between diagnosis and recurrence, therapy and outcome in terms of CAAs. The selected articles were reviewed by two independent authors, and judged on their relevant contribution to the subject of the study. The Preferred Reporting Items for Systematic Review And Meta-Analysis (PRISMA) guidelines were followed.

### Quality assessment

The quality of the included studies was evaluated through the Quality Appraisal Checklist for Case Series Studies of IHE (excluding not applicable items) [13] and through Checklist for Case Reports of The Joanna Briggs Institute Critical Appraisal tools [14] (see Tables 1 and 2 of supplemental materials).

## Results

After an extensive search in Pubmed and Scopus, 30 papers were found, an additional hand search of the available bibliography provided 5 more papers, so a total of 35 papers were found.

**Fig. 1:**
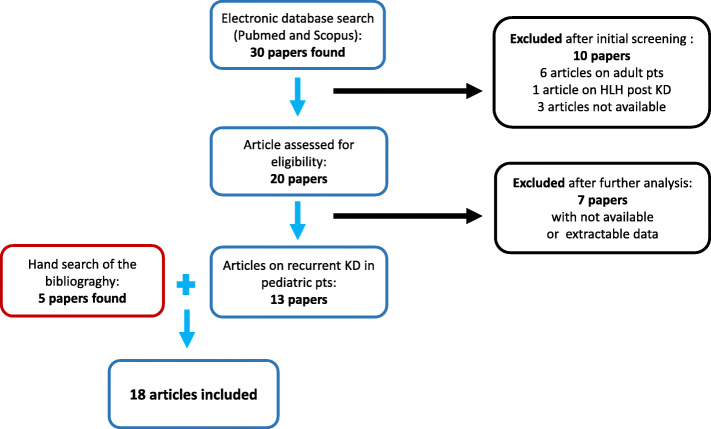
Search strategy for the systematic review

Of these papers, 10 were excluded after initial screening of abstract and title: 6 papers regarding recurrent KD in adult patients, 1 paper regarding hemophagocytic lymphohistiocytosis following KD, 3 because article was not available. Other 7 papers were excluded because clinical and laboratory data were not available or extractable. So a total of 17 papers were excluded from analysis. See Fig. 1.

Eventually a total of 18 articles dating from 1993 to 2019 and reporting 105 cases of paediatric recurrent KD were included in our review. Most articles were single case reports [2, 4, 15–25], while 5 were case series of respectively 20 [26], 22 [27], 14 [28], 25 [3], 7 [29] patients. We analyzed anamnestic and clinical data of 106 patients (including our new case). Data regarding age and sex, number of recurrences, interval elapsed between diagnosis and recurrences, IVIG administration at diagnosis and at recurrence, CAAs at diagnosis and at recurrence, are reported in Table 1.

**Table 1:** Data regarding age and sex, number of recurrences, interval elapsed between diagnosis and recurrences, IVIG administration at diagnosis and at recurrence, CAAs at diagnosis and at recurrence

Author ^**ref**^. Nation (notes)	Num. of pts	Age at first recurrence (months)	Sex	Months between first diagnosis and recurrence	Num. Of recurrences	Num. Of pts receiving IVIG at D	Num. of pts receiving IVIG at recurrence	Num. of pts with CAA at first diagnosis	Num. of pts with CAA at recurrence
Guleria et al. [3] India (Case series)	7	Mean 36^*^	M 5^#^	8.7 ^*^	1	6	5	1	1
			F 2^#^						
Goswami et al. [2] USA	1	120	F	49 (1th)	2	na	1	0	1
				109 (2th)					
Verma et al. [4] India	1	96	F	48	1	1	1	Na	1
Kang et al. [27] Korea (Case series)	25	Mean 49^*^	M 16^#^	21 (1th)	1; 22^#^	25	25	5	2
				Na (2th)	2; 2^#^				
			F 9^#^						
				Na (3th)	3; 1^#^				
Saha et al. [13] India	1	96	M	24	1	0	1	1	1
Lee et al. [26] Korea (Case series)	14	Mean 41^*^	M 8^#^	18^*^	1	Na	Na	Na	Na
			F 6^#^						
Verma et al. [14] India	1	8	F	48	1	Na	1	Na	Na
Aldemir Kocabaş et al. [15] Turkey	1	108	M	90	1	1	1	1	0
See et al. [16] Singapore	1	48	M	30	1	1	1	1	1
Yang et al. [28] China (Case series)	22	Mean 30^*^	M 9^#^	12^*^	1	22	22	10	6
			F 13^#^						
Osman et al. [17] Sudan	1	72	M	64	1	1	1	0	1
Balasubramanian et al. [18] India	1	16	M	11	1	1	1	0	1
Zou et al. [24] China (Case series)	20	Na	na	15^*^	Na	Na	Na	5	8
Pemberton et al. [20] UK	1	72	M	15	1	1	1	0	0
Matsubara et al. [21] Japan	1	68	F	38	1	0	1	1	1
Nakada et al. [19] Japan (Case series)	5	Na	Na	Na	1	Na	Na	Na	1
Nigro et al. [22] Italy	1	10	F	1	1	0	0	0	Na
Hamada et al. [23] Japan	1	12	M	1 (1th)	3	1/1	1	0	0
				3 (2th)					
				12 (3th)					
Present case Italy	1	29	M	9 (1th)	2	1	1	0	0
				19 (2th)					

^*^ average value in months. ^#^ Num. of pts.; *Ref* reference, *num* number, *pts.* patients, *D* diagnosis, *IVIG* intravenous immunoglobulins, *CAA* coronary artery abnormalities

Where data about age and sex were available, the average age at first recurrence was 58 months, 45/106 children were boys, 36/106 girls with a male to female ratio of 1:0.8. Of the 106 patients 80 had just 1 recurrence, 4 had 2 recurrence, 2 had 3 recurrence. Median time elapsed between diagnosis and first recurrence was 33 months. For children with more than one recurrence, median time elapsed between diagnosis and II recurrence was 43.6 months and between II and III recurrence it was 12 m. Concerning IGIV therapy where the information was available, 61/106 patients (57,5%) received IVIG at first episode, 4 did not (4%); of the 61 who received IVIG at diagnosis 60 (98%) received IVIG also at recurrences. The total of patients receiving IVIG at recurrence was 64/106 (60%), 3/106 (3%) did not. Twenty-five patients (23.5%) had CAAs at first episode, 59 had no CAAs (55.5%), for 21 (20%) patients this data is not available (NA). Of the 25 with CAAs at first diagnosis, 20 had CAAs (80%) also at the recurrence.

At recurrence 25 patients had CAAs (23.5%), 64 (60%) had not, for 16 patients (15%) this data is NA. Of the 25 patients with CAAs at recurrence, 3 (12%) had not CAAs at first diagnosis, while for 2 (8%) this data is NA. For all patients duration of follow up was variable or not reported.

## Discussion

KD currently is the leading cause of acquired heart disease in childhood in developed countries [1] Cardiovascular complications are the major cause of morbidity and responsible for virtually all deaths from KD. It’s known that KD can recur but there is a paucity of literature on recurrence rates of KD. The proportion of recurrent cases among children with a history of KD varies among countries: 3–4% in Japan [30, 31], 3.8% in Korea [32], 1.9% in China [27],1.5% in Taiwan [33], 3.5% in Jamaica [34], 1.7% in the USA [35] and 1.5% in Canada [36] In USA rates of recurrent KD are higher in children of Asian descent compared to the Caucasian population [35]. Sudo et al. [30] in their nationwide survey in Japan compared incidence rates of recurrent KD between different years, confirming that rates between 2003 and 2012 didn’t change compared with those of 1980s–90s. Moreover, they [30] found that rate for recurrence was statistically higher among males, ≤ 3 years old and those who received intravenous immunoglobulin at the initial episode, while the presence of cardiac sequelae during the initial episode did not affect the recurrence incidence. They defined as risk factors for recurrent KD male sex, young age and initial resistance to immunoglobulin therapy [30] Also Guleria et al. [3] and Kang et al. [29] reported a male predominance in KD recurrence, while Maddox et al. [37] found that KD patients with recurrent episodes were more likely to be older, have atypical presentations, and CAAs regardless of previous IVIG therapy. Recurrent KD more than 2 y from diagnosis is rarely reported, and multiple recurrences are rarely described [17, 29, 37, 38] .

KD, as well as its recurrences, has unknown etiology, but it has been supposed that diverse infectious agents (including coronavirus) can trigger a “final common pathway” of immune dysregulation causing KD in a genetically susceptible individual [39]

Both bacteria and viruses have been sporadically isolated from KD patients and their proteins acting as superantigens proposed as possible triggers of a dysregulated immune response. Between viruses, Epstein Barr virus, adenovirus, parvovirus B19, herpesvirus 6, parainfluenza type 3, measles, rotavirus, dengue virus, and human immunodeficiency virus have been reported as the most frequently associated with KD. VZV, 2009 H1N1 pandemic influenza, Coxsackie B3 virus, human bocavirus [40] and human coronavirus (HCoV) NL63 [41] and HCoV229E [42] have also been described in patients with KD. The recent finding of intracytoplasmic inclusion bodies in tissues from patients with KD [43, 44] and RNA virus-like inclusion bodies in the cytoplasm of bronchoepithelial cells of KD patients [45, 46] supports the thesis that RNA viruses are closely involved in the etiopathogenesis of KD.

Furthermore, there are growing evidences that superantigens, a family of proteins able to cause a dramatic T cell dependent immune activation, are involved in the aetiology of KD [47] and recently it has been demonstrated that Sars-Cov-1 viral proteins could act as superantigens/toxins [48] triggering a dysregulated immune response [40]

Therefore, in our case SARS-CoV-2 infection with the related hyperinflammation in COVID-19 could have been the trigger leading to KD recurrence through the endothelial dysfunction via endothelial ACE2 [6, 49]

Of the 18 article of recurrent KD we included in the analysis just Nigro et al. [16] report parvovirus B-19 as clear etiological agent of the recurrent episode in an HIV-1 infected child. In the other papers no clear infectious etiology is reported, while Hamada et al [17] conclude that Streptococcus spp. just might be associated with onset of recurrent KD.

In many paediatric Covid-19 cases a cytokine storm has been documented [7–12, 50], which required the definition of the new nosological entity of multisystem inflammatory syndrome of children (MISC) temporally associated with Sars-Cov2 [51–53] whose pattern of overexpressed cytokines overlaps with that typical of KD [5]. MISC can present with prevalent abdominal symptoms with excessive inflammatory markers [54] like in atypical KD, in which abdominal pain is documented and can be significant enough to prompt advanced imaging and surgical consultation [55]

Even if some features of MISC overlapped with that of KD and KD shock syndrome [50], there are marked epidemiologic differences which make it clear that the 2 conditions are not the same [39]: MISC generally occurs in older children (median age 9–10 years in largest series [50]) of African descent [10, 54], while KD predominantly occurs in children ≤5 years of age [11, 50, 56, 57] of Asian origin [6]

Moreover, they differ even for some clinical and laboratory features: CAAs are typical in KD, while they are rare and often transitory in MISC, in which other organs involvement prevails, such as pulmunar or abdominal districts; in MISC higher levels of C-reactive protein, ferritin [50], fibrinogen, D-dimer [12] are documented, plus lymphopenia. In case of MISC with cardiac involvement, compared to KD, higher levels of markers of cardiac injury, like troponin and NT-proBNP are documented, often with depression of ventricular function [12], valve regurgitation and pericardial effusions [7, 9–11], which are not characteristic of KD [9, 50, 58–60]

Although clinical characteristics of KD and MISC partly overlap, we think our case’s diagnosis was recurrent KD instead of MISC because epidemiological (male, 3 y old with a previous history of KD treated with IVIG), clinical (no abdominal pain, none of MISC-typical echocardiographic findings) and laboratory features (normal AST/ALT, LDH, pro-BNP and ferritin, no lymphopenia) were suggestive of KD rather than MISC. Also, considering clinical picture, rapid and full response to IVIG, negativity of other microbiological and rheumatological exams, plus no symptoms recrudescence, other diagnosis (including autoinflammatory and genetic diseases) were reasonably excluded.

This study has many limitations. Regarding the case report: we retrospectively analyzed it as we admitted the patient before the first NHS England alert on this emerging multisystem inflammatory disease Covid-19 related and, than, before the case definition of MISC.

Regarding materials and methods: we performed a computerized search using just 2 databases (Pubmed and Scopus), plus we hand-searched through references ‘revision of included papers, but some articles of grey literature could have been missed.

Regarding papers included in the review: they are heterogenous, as some are case reports and other case series and not all describe in detail microbiological and immunological tests performed in the diagnostic work up. Also from quality assessment a low risk of bias emerged for all the included case reports, while a low-moderate risk of bias emerged for the included case series, being their main limitations the retrospective nature of the studies and the absence of a clear follow up.

## Conclusions

We believe SARS Cov2 can act as a trigger capable to determine, in a genetically susceptible individual, a KD recurrence. Fortunately, patients with KD and MIS-C both improve with IVIG and corticosteroid therapy, and in case of failure a second dose of IVIG or TNF-α or IL-1 inhibitor can be considered, although the efficacy of any therapy for MIS-C is evolving and yet unproven [39]

Reporting this case we want to build evidences on the clinical correlation between SARS-Cov-2 and KD in pediatric patients and stress the paramount importance for KD recurrences in COVID-19 era to look for a SARS Cov2 infection.

## Supplementary Information

**Additional file 1: Supplemental materials. Table 1** Quality Appraisal Checklist for Case Series Studies of IHE. **Supplemental materials. Table 2** Checklist for Case Reports of The Joanna Briggs Institute Critical Appraisal tools.

## Data Availability

All data used and/or analysed during this study are included in this published article.

## Abbreviations

KD: Kawasaki disease
CAAs: Coronary artery abnormalities
Sars-Cov-2: Severe acute respiratory Syndrome Coronavirus 2
IVIG: Intravenous immunoglobulin
NA: Not available
COVID-19: Coronavirus disease 19
MISC: Multisystem inflammatory syndrome of children
P-MIS: Pediatric multisystem inflammatory syndrome
