# Supplementary material for: Kawasaki disease recurrence in the COVID-19 era: a systematic review of the literature
Source: Ital J Pediatr. 2021 Apr 19;47:95. doi: 10.1186/s13052-021-01041-4 (PMC8054252; doi:10.1186/s13052-021-01041-4)
Supplement: Supplementary file 1 — Additional file 1: Supplemental materials. Table 1 Quality Appraisal Checklist for Case Series Studies of IHE. Supplemental materials. Table 2 Checklist for Case Reports of The Joanna Briggs Institute Critical Appraisal tools. [file 13052_2021_1041_MOESM1_ESM.docx]

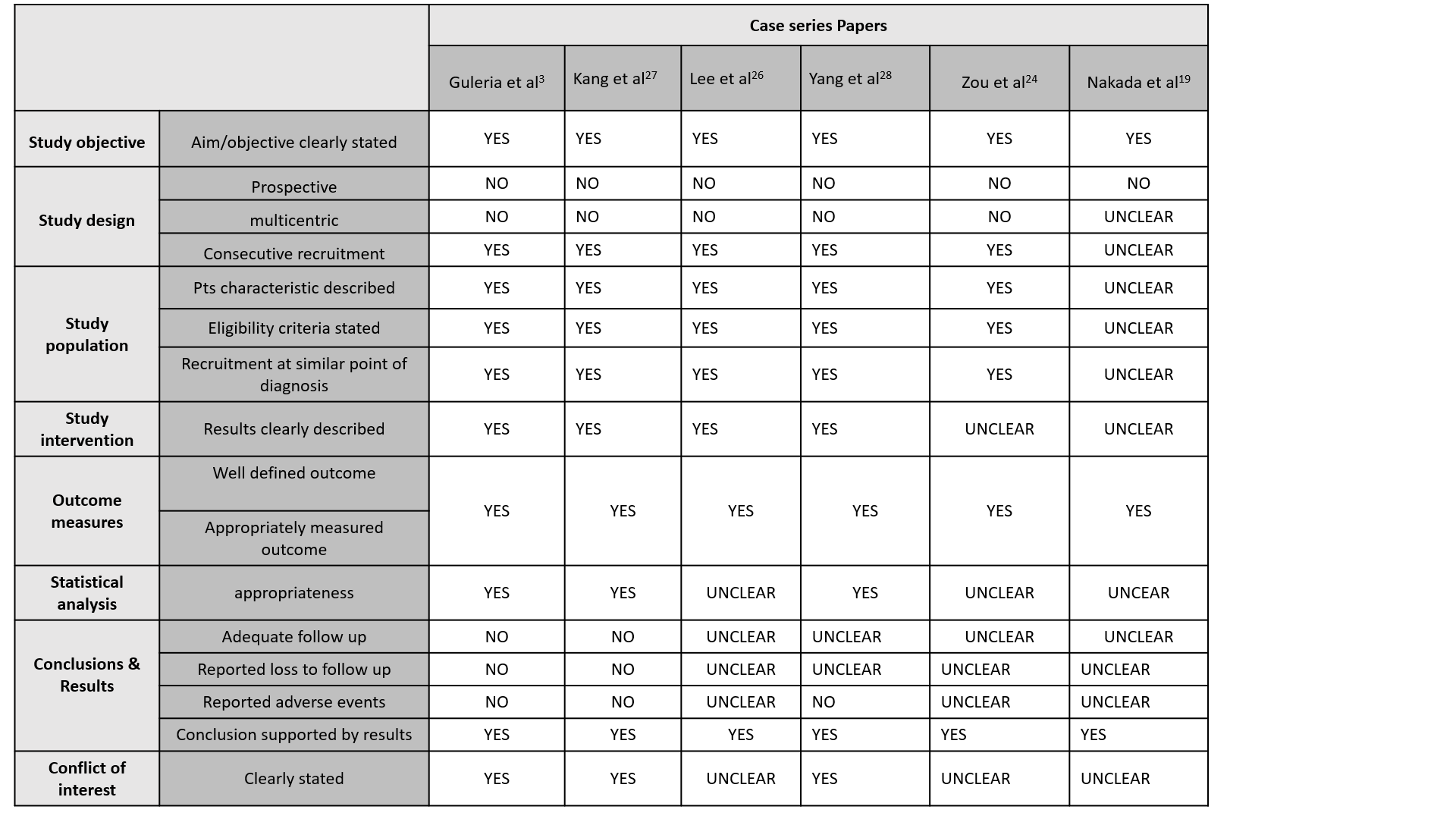


Supplemental materials. Table 1 - Quality Appraisal Checklist for Case Series Studies of IHE.


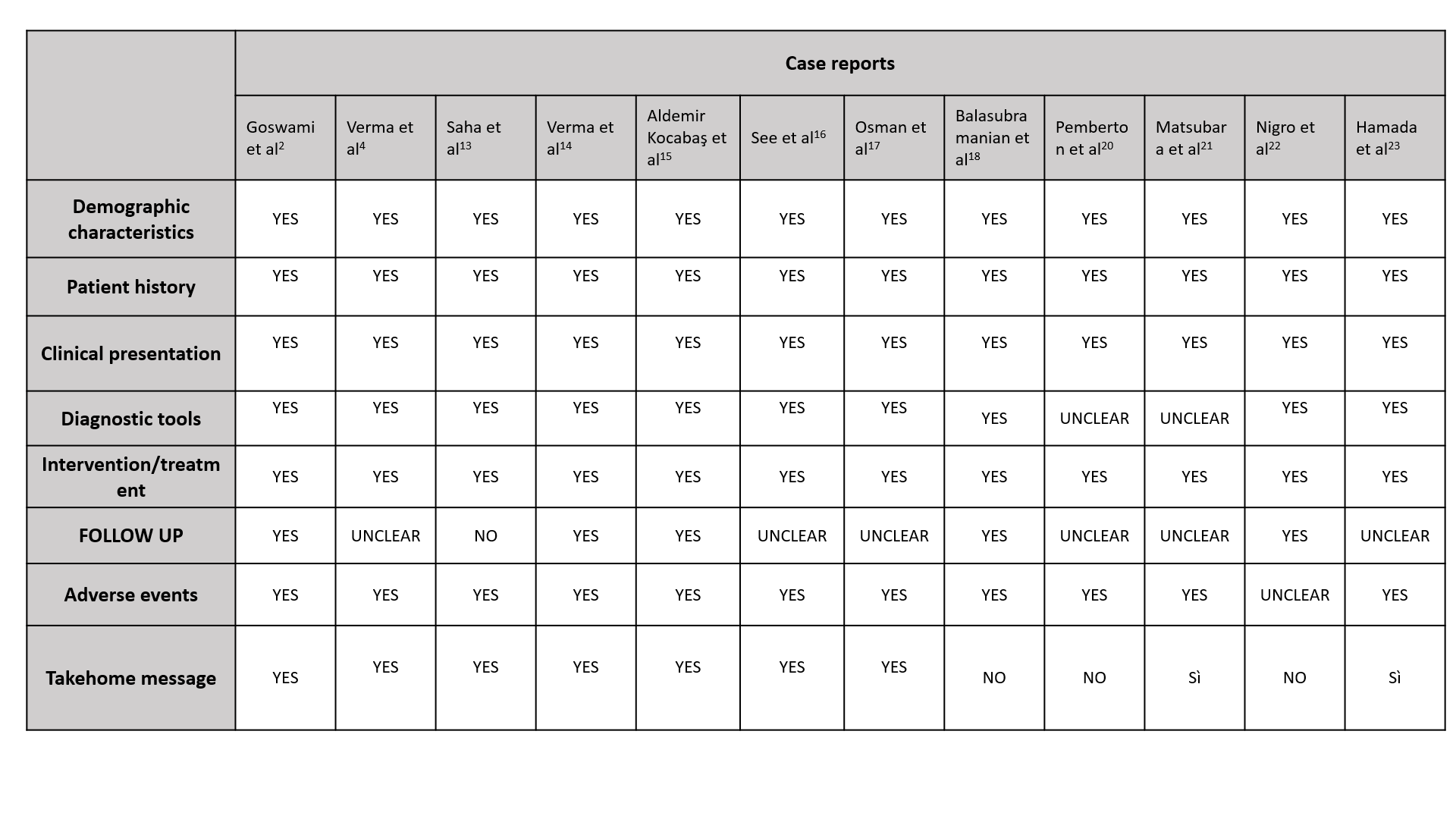


Supplemental materials. Table 2 - Checklist for Case Reports of The Joanna Briggs Institute Critical Appraisal tools.
